# Defects, Dopants and Sodium Mobility in Na_2_MnSiO_4_

**DOI:** 10.1038/s41598-018-32856-7

**Published:** 2018-10-02

**Authors:** Navaratnarajah Kuganathan, Alexander Chroneos

**Affiliations:** 1 Department of Materials, Imperial College London, London, SW7 2AZ United Kingdom; 2 Faculty of Engineering, Environment and Computing, Coventry University, Priory Street, Coventry, CV1 5FB United Kingdom

## Abstract

Sodium manganese orthosilicate, Na_2_MnSiO_4_, is a promising positive electrode material in rechargeable sodium ion batteries. Atomistic scale simulations are used to study the defects, doping behaviour and sodium migration paths in Na_2_MnSiO_4_. The most favourable intrinsic defect type is the cation anti-site (0.44 eV/defect), in which, Na and Mn exchange their positions. The second most favourable defect energy process is found to be the Na Frenkel (1.60 eV/defect) indicating that Na diffusion is assisted by the formation of Na vacancies via the vacancy mechanism. Long range sodium paths via vacancy mechanism were constructed and it is confirmed that the lowest activation energy (0.81 eV) migration path is three dimensional with zig-zag pattern. Subvalent doping by Al on the Si site is energetically favourable suggesting that this defect engineering stratergy to increase the Na content in Na_2_MnSiO_4_ warrants experimental verification.

## Introduction

Sodium ion batteries have attracted attention as a dominant power source in large scale energy storage applications due to the low cost and high abundance of sodium as compared to lithium^1–3^.

New class of cathode materials providing large quantity of Na ions in sodium batteries can lead to the high power density and high energy density. Several promising sodium based cathode materials such as NaFePO_4_^4–6^, Na_2_FePO_4_F^7,8^, Na_3_V_2_(PO_4_)_3_^9,10^ and Na_x_TMO_2_ (TM = Ti, V, Cr, Mn, Fe, Co and Ni)^11–13^ were reported in the experimental studies. Discovery of novel cathode materials is still active to produce high capacity battery to satisfy the growing demand.

Orthosilicate Na_2_MnSiO_4_ has been recently proposed as a promising Na storage material because of its impressive sodium storage performance, low cost and environmentally benign^14–19^. The structural stability of this material is mainly provided by the (SiO_4_)^4−^ matrix via strong Si-O bonds. Further, it may be possible to extract more than one sodium more readily from Na_2_MnSiO_4_ since Mn can form the Mn^4+^ oxidation state. Chen *et al*.^15^ synthesized Na_2_MnSiO_4_ by a sol-gel method and reported a reversible capacity of 125 mAhg^−1^ at a rate of C/10 using an ionic liquid electrolyte at elevated temperatures. Law *et al*.^14^ prepared Na_2_MnSiO_4_ *via* a modified two-step route and reported an impressive sodium storage performance of 210 mAhg^−1^ at 0.1 C. Using first-principles calculations, Zhang *et al*.^19^ investigated ion diffusion mechanism of Na_2_MnSiO_4_ and concluded that the Na ion diffusion is faster than Li ion diffusion in Li_2_MnSiO_4_. There are a limited number of experimental and theoretical work reported in the literature.

As experiments cannot provide detailed information about the defects and Na ion diffusion paths with activation energies, classical modelling techniques are widely used to calculate those properties. This theoretical approach has been successfully applied on a wide range of lithium ion battery materials and a few sodium ion battery materials^20–25^. Very recently, we have applied this simulation technique to examine the defect chemistry, lithium transport and the effect of dopants on lithium vacancy formation on the Li_5_FeO_4_^26^, Li_2_CuO_2_^27^ and Li_9_V_3_(P_2_O_7_)_3_(PO_4_)^28^. The present study uses atomistic modeling techniques to calculate the energetics for the formation of defects, solution of trivalent dopants and Na ion diffusion paths in Na_2_MnSiO_4_.

## Results and Discussion

### Na_2_MnSiO_4_ structure

**Figure 1:**
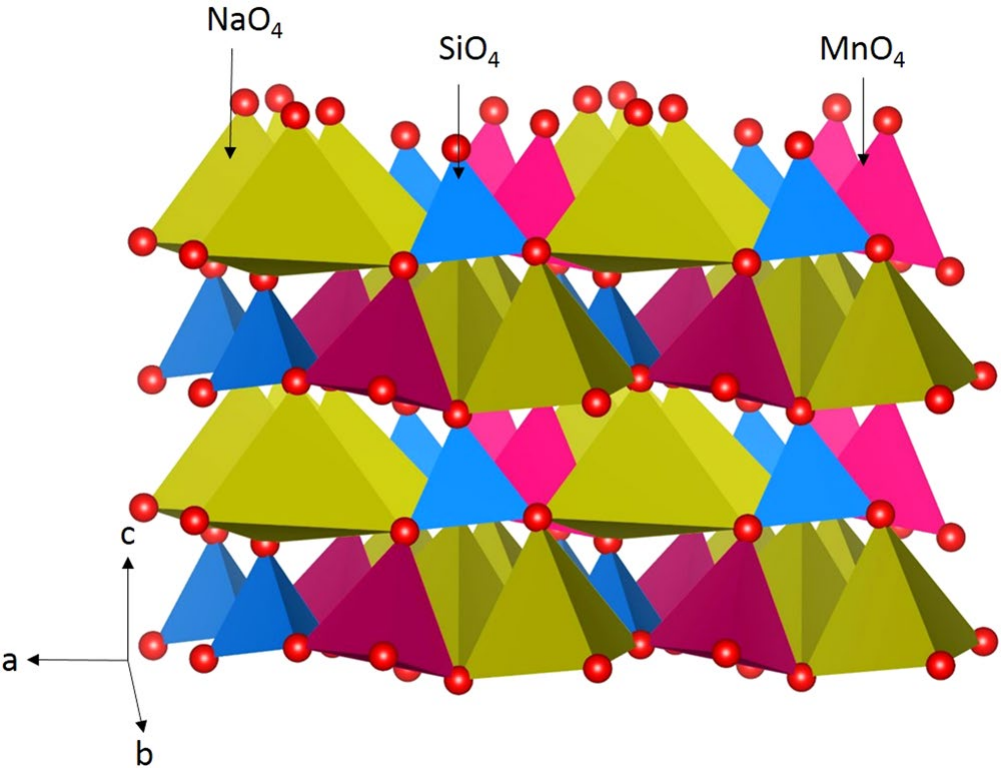
Crystal structure of Na_2_MnSiO_4_ (space group P*n*).

The crystal structure of Na_2_MnSiO_4_ exhibits a monoclinic crystallographic structure with space group *Pn* (lattice parameters a = 7.02857 Å, b = 5.60957 Å, c = 5.33391 Å, α = 90.0°, β = 89.7949° and γ = 90°) as reported by Nalbandyan *et al*.^16^ In the Fig. 1, crystal structure of Na_2_MnSiO_4_ is shown together with bonding nature of all cations forming corner-sharing tetrahedral units with four O atoms. First, experimental monoclinic crystal structure was reproduced using classical pair potentials as tabulated in Table S1 in the Supplementary Information. There is an excellent agreement between experimental and calculated equilibriumlattice constants (refer to Table 1).

**Table 1:** Calculated and Experimental Structural Parameters for Monoclinic (P*n*) Na_2_MnSiO_4_.

Parameter	Calc	Expt^13^	|∆| (%)
a (Å)	6.995595	7.028570	0.47
b (Å)	5.644977	5.609570	0.63
c (Å)	5.334769	5.333910	0.02
α (°)	90.0000	90.0000	0.00
β (°)	89.7683	89.7949	0.03
γ (°)	90.0000	90.0000	0.00

### Intrinsic defect processes

Next we calculated different isolated point defects including vacancies and interstitials to calculate the Frenkel and Schottky-type defect formation energies in Na_2_MnSiO_4_. These intrinsic defect energetics can provide useful information about the electrochemical behavior of Na_2_MnSiO_4_. Here we write equations for the Frenkel, Schottky and anti-site defect formation using the Kröger-Vink notation^29^.

1 $${\rm{Na}}\,{\rm{Frenkel}}:{{\rm{Na}}}_{{\rm{Na}}}^{{\rm{X}}}\to {V}_{{\rm{Na}}}{{\prime} }+{\,\mathrm{Na}}_{{\rm{i}}}^{\bullet }$$

2 $${\rm{Mn}}\,{\rm{Frenkel}}:{{\rm{Mn}}}_{{\rm{Mn}}}^{{\rm{X}}}\to {V}_{{\rm{Mn}}}{{\prime} {\prime} }+{\,\mathrm{Mn}}_{{\rm{i}}}^{\bullet \bullet }$$

3 $${\rm{O}}\,{\rm{Frenkel}}:{{\rm{O}}}_{{\rm{O}}}^{{\rm{X}}}\to {V}_{{\rm{O}}}^{\bullet \bullet }+{{\rm{O}}}_{{\rm{i}}}{{\prime} {\prime} }$$

4 $${\rm{Si}}\,{\rm{Frenkel}}:{{\rm{Si}}}_{{\rm{Si}}}^{{\rm{X}}}\to {V}_{{\rm{Si}}}{{\prime} {\prime} {\prime} {\prime} }+{\,\mathrm{Si}}_{{\rm{i}}}^{\bullet \bullet \bullet \bullet }$$

5 $${\rm{Schottky}}:2\,{{\rm{Na}}}_{\mathrm{Na}\,}^{{\rm{X}}}+{{\rm{Mn}}}_{{\rm{Mn}}}^{{\rm{X}}}+{{\rm{Si}}}_{{\rm{Si}}}^{{\rm{X}}}+4\,{{\rm{O}}}_{{\rm{O}}}^{{\rm{X}}}\to 2\,{V}_{{\rm{Na}}}{{\prime} }+{V}_{{\rm{Mn}}}{{\prime} {\prime} }+{V}_{{\rm{Si}}}{{\prime} {\prime} {\prime} {\prime} }+4{V}_{{\rm{O}}}^{\bullet \bullet }+{{\rm{Na}}}_{2}{{\rm{MnSiO}}}_{4}$$

6 $${{\rm{Na}}}_{2}{\rm{O}}\,{\rm{Schottky}}:2\,{{\rm{Na}}}_{{\rm{Na}}}^{{\rm{X}}}+{{\rm{O}}}_{{\rm{O}}}^{{\rm{X}}}\to 2\,{V}_{{\rm{Na}}}{{\prime} }+{V}_{{\rm{O}}}^{\bullet \bullet }+{{\rm{Na}}}_{2}{\rm{O}}$$

7 $${\rm{MnO}}\,{\rm{Schottky}}:{{\rm{Mn}}}_{{\rm{Mn}}}^{{\rm{X}}}+{{\rm{O}}}_{{\rm{O}}}^{{\rm{X}}}\to {V}_{{\rm{Mn}}}{{\prime} {\prime} }+{V}_{{\rm{O}}}^{\bullet \bullet }+{\rm{MnO}}$$

8 $${{\rm{SiO}}}_{2}\,{\rm{Schottky}}:{{\rm{Si}}}_{{\rm{Si}}}^{{\rm{X}}}+2\,{{\rm{O}}}_{{\rm{O}}}^{{\rm{X}}}\to {V}_{{\rm{Si}}}{{\prime} {\prime} {\prime} {\prime} }+2\,{V}_{{\rm{O}}}^{\bullet \bullet }+{{\rm{SiO}}}_{2}$$

9 $${\rm{Na}}/{\rm{Mn}}\,{\rm{antisite}}\,({\rm{isolated}}):{{\rm{Na}}}_{{\rm{Na}}}^{{\rm{X}}}+{{\rm{Mn}}}_{{\rm{Mn}}}^{{\rm{X}}}\to {\mathrm{Na}}_{{\rm{Mn}}}{{\prime} }+{{\rm{Mn}}}_{{\rm{Na}}}^{\bullet }$$

10 $${\rm{Na}}/{\rm{Mn}}\,{\rm{antisite}}\,({\rm{cluster}}):{{\rm{Na}}}_{{\rm{Na}}}^{{\rm{X}}}+{{\rm{Mn}}}_{{\rm{Mn}}}^{{\rm{X}}}\to {\{{\mathrm{Na}}_{{\rm{Mn}}}{{\prime} }:{{\rm{Mn}}}_{{\rm{Na}}}^{\bullet }\}}^{{\rm{X}}}$$

**Figure 2:**
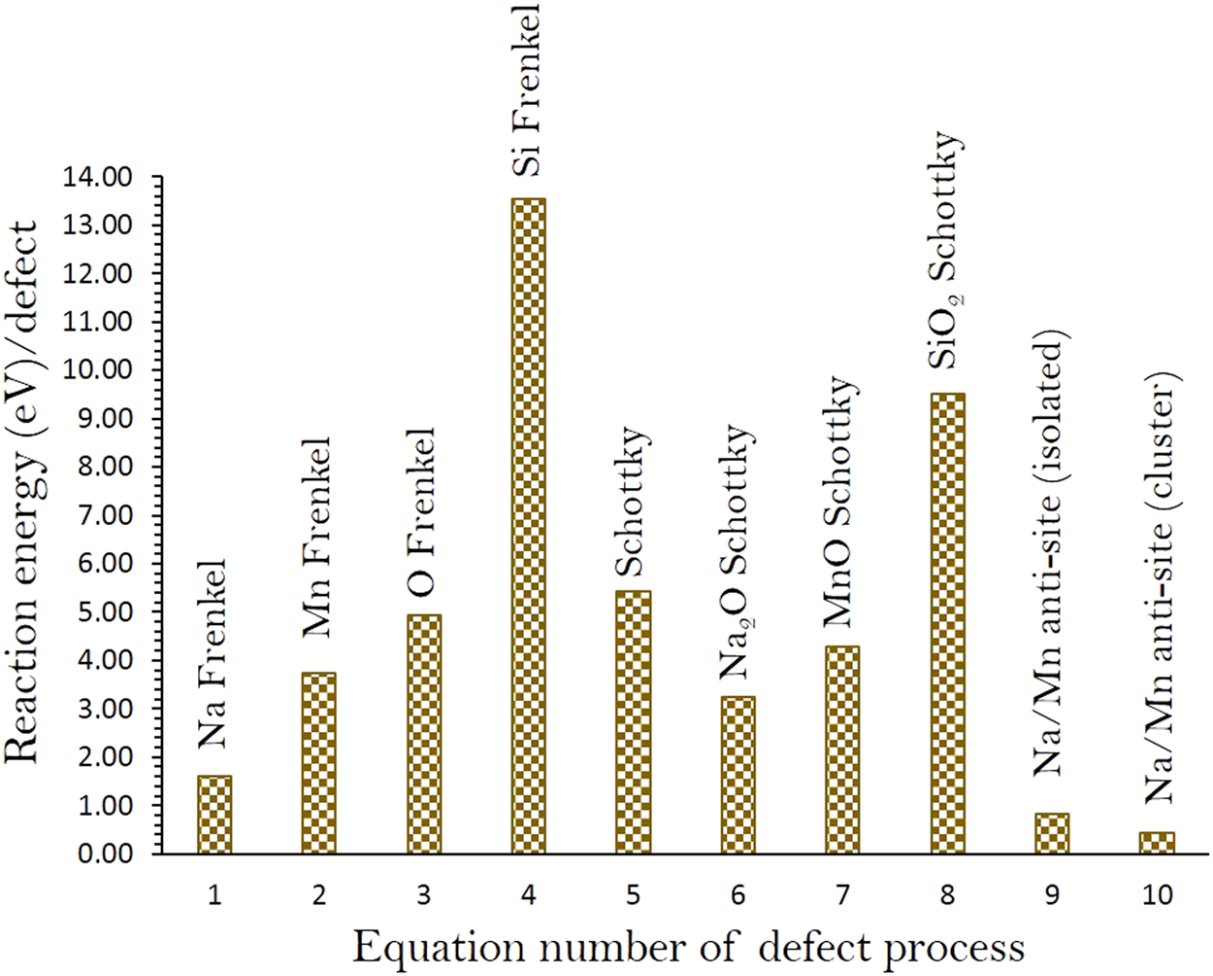
Energetics of intrinsic defect process in monoclinic Na_2_MnSiO_4_.

Figure 2 shows the reaction energies for these intrinsic defect processes (refer to Table S2 for the energies). Na-Mn anti-site was calculated to be the most favorable intrinsic disorder meaning that at high temperatures a small percentage of Na on Mn sites (${\mathrm{Na}}_{{\rm{Mn}}}{{\prime} }$) and Mn on Na sites (${{\rm{Mn}}}_{{\rm{Na}}}^{\bullet }$) will be observed. In the relaxed defect structure, a small amount of distortion is observed in the cation-oxygen bond lengths and bond angles, but overall structure of the lattice was not distorted significantly. This type of defect has been observed experimentally in different class of Li ion cathode battery materials during cycling and theoretically in some as prepared Na ion cathode materials^23–25,30–35^. The Na Frenkel was found to be the second most favourable defect process. The Frenkel and Schottky defect energies were calculated to be highly endothermic meaning that they are unfavorable. The formation enthalpy of Na_2_O Schottky (relation 6) is 3.25 eV per defect (refer to Table S2, Supplementary Information). At elevated temperatures, this process can take place to form further ${V}_{{\rm{Na}}}{{\prime} }$ and ${V}_{O}^{\bullet \bullet }$.

### Sodium ion-diffusion

**Figure 3:**
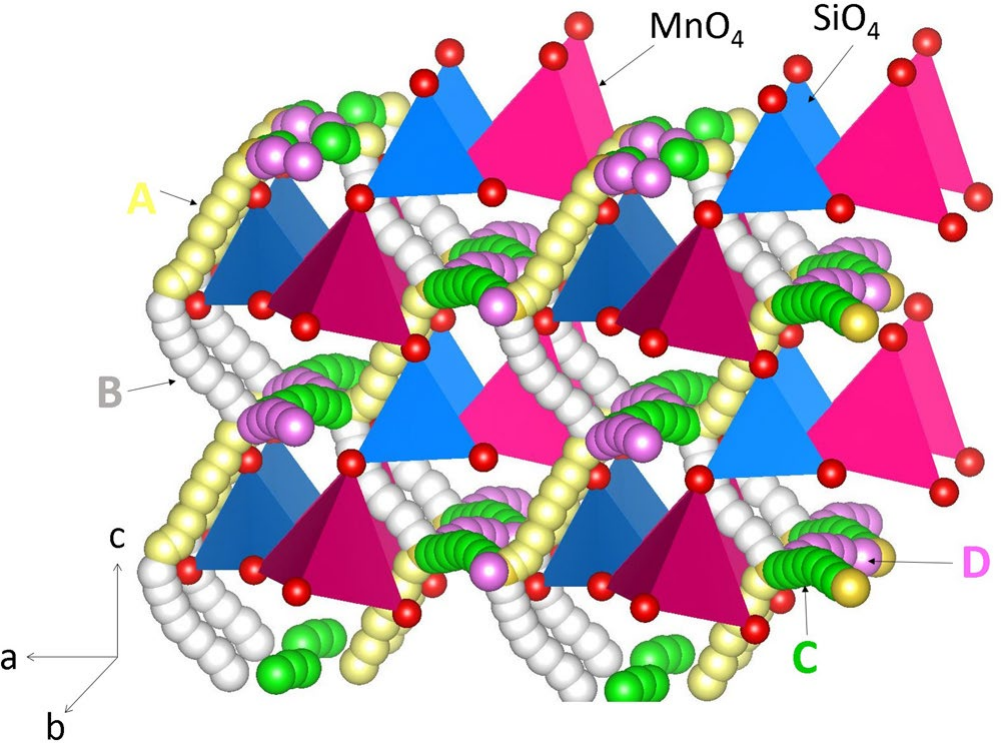
Possible long range sodium vacancy migration paths considered. Local Na migration paths are shown in green, yellow, white and purple atoms. SiO_4_ and MnO_4_ tetrahedral units are shown blue and pink colors respectively.

**Figure 4:**
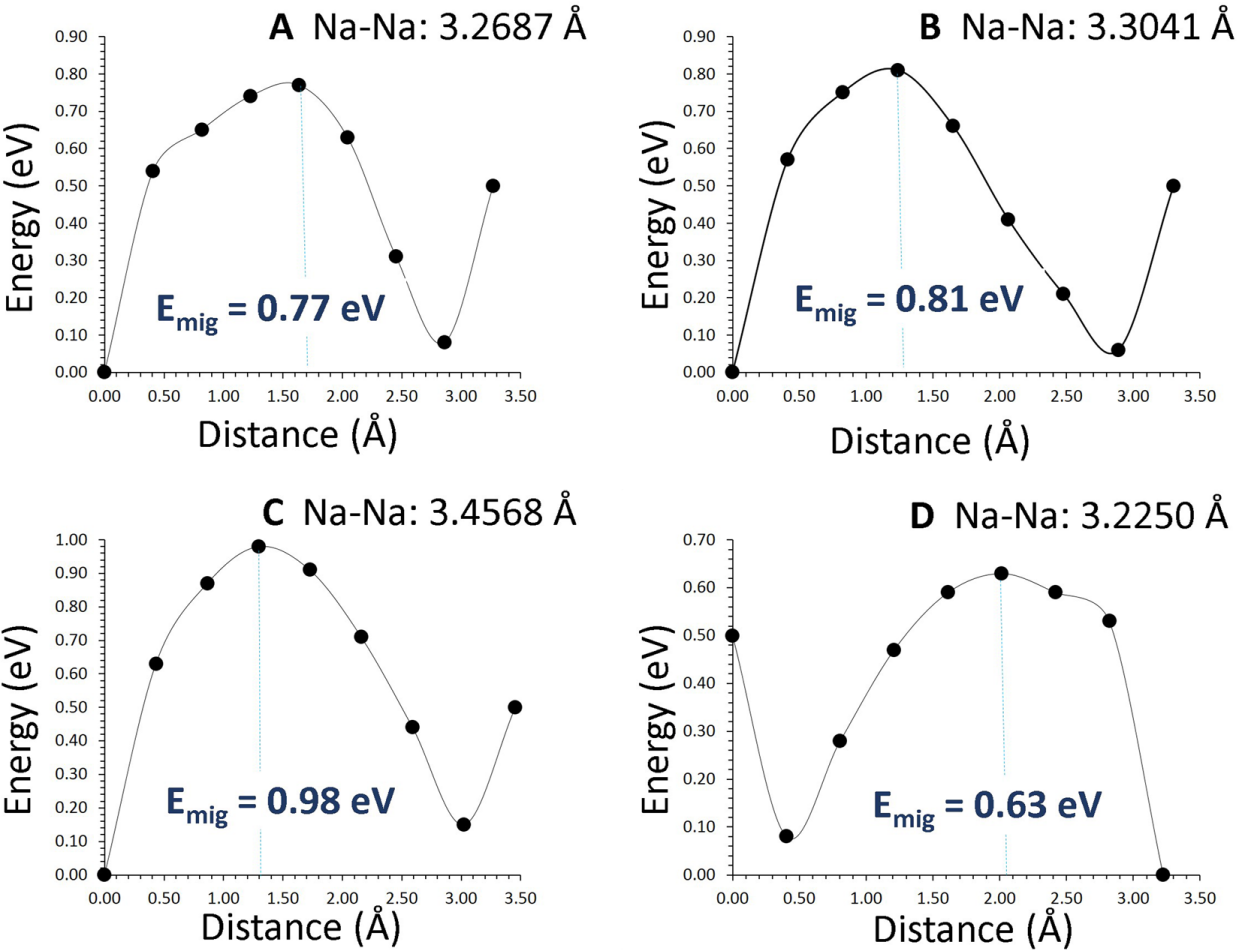
Four different energy profiles [as shown in Fig. 3] of Na vacancy hopping between two adjacent Na sites in Na_2_MnSiO_4_.

The intrinsic sodium ion diffusion of Na_2_MnSiO_4_ is discussed in this section. Activation energy of Na ion diffusion together with diffusion paths are important when Na_2_MnSiO_4_ is assessed as a potential material in sodium ion batteries. The current computaional technique enables the calculation of Na diffusion paths together with activation energies, which are diffcult to investigate by experiments. For the Na vacancy migration, four Na local hops (A, B, C and D) were identified. Hop A is between two Na sites with the jump distance of 3.2687 Å and the migration energy is calculated to be 0.77 eV (refer to Table 2). The migration path for hop A is in the *ac* plane and Na ion moves via a curved trajectory. In the hop B, Na ions diffuses in the *ac* plane with a curved trajectory but the jump distance of 3.3041 Å and migration energy of 0.81 eV which are different from those calculated for hop A. Hops C and D are in the *ab* plane with each forming curve trajectories. Their jump distances are 3.4568 Å and 3.3350 Å with corresponding migration energies of 0.98 eV and 0.63 eV respectively. Two possible three dimensional lower energy long range paths (A → B → A → B) and (C → D → C → D) connecting local Na hops were identified as shown in Fig. 3. These two paths exhibited zig-zag pattern with overall activation energies of 0.81 eV and 0.98 eV respectively. We considered other possible long range paths connecting local Na hops. However, the lowest overall activation energy was calculated to be 0.81 eV. Figure 4 reports the energy profile diagrams for Na local hops with activation energies. Zhang *et al*.^19^ calculated the ion diffusion mechanism in Li_2_MnSiO_4_ and Na_2_MnSiO_4_ using density functional theory and concluded that Na^+^ migration has relatively lower activation energy barrier than that of Li^+^ migration. In our previous modelling for monoclinic Li_2_MnSiO_4_, the lowest over all activation energy for Li ion migration is 1.58 eV^21^. In the current study, the lowest overall Na ion migration barrier is calculated to be 0.81 eV much lower than that found for Li ion migration in agreement with the study of Zhang *et al*.^19^. Furthermore, in their study, Na^+^ ion migrate *via* three dimensional channels and the overall activation energy is calculated to be 0.54 eV. The activation energy difference is due to two different methodologies. Also density functional theory (DFT) is constrained by finite size effects. Small system sizes are a well-known source of error in DFT calculations. Furthermore, in the present study, ions were treated as fully charged as point defects in a highly ionic material might be expected to be in their fully ionic charge states. The position of the highest potential energy along the migration path is defined as the activation energy of migration.

**Table 2:** Calculated Na-Na separations and activation energies for the sodium ion migration between two adjacent Na sites refer to Fig. 3.

Migration path	Na-Na separation (Å)	Activation energy (eV)
A	3.2687	0.77
B	3.3041	0.81
C	3.4568	0.98
D	3.2250	0.63

### Trivalent doping

Incorporation of additional sodium into the as-prepared material will enhance the capacity and further increase the applicability of Na_2_MnSiO_4_ as a potential cathode material for sodium ion batteries. A possible approach to incorporate additional Na is by doping trivalent cations on Si site through creating Na interstitials. Similar approach has been previously demonstrated in Li_2_MnSiO_4_ cathode material^21^. Here we considered the solution of *R*_2_*O*_3_ (*R* = Al, Ga, Sc, In, Y, Gd and La) via the following process (in Kröger-Vink notation):

11 $${{\rm{R}}}_{2}{{\rm{O}}}_{3}+2\,{{\rm{Si}}}_{{\rm{Si}}}^{{\rm{X}}}+{{\rm{Na}}}_{2}{\rm{O}}\to 2\,{{\rm{R}}}_{{\rm{Si}}}{{\prime} }+2\,{{\rm{Na}}}_{{\rm{i}}}^{\bullet }+2\,{{\rm{SiO}}}_{2}$$

**Figure 5:**
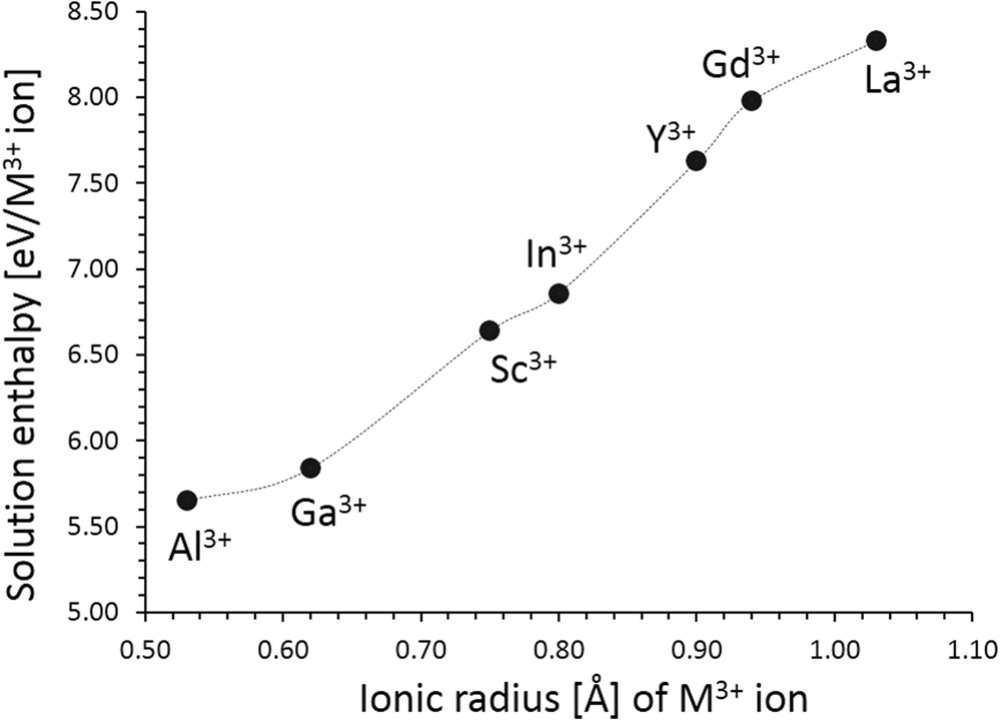
Enthalpy of solution of R_2_O_3_ (*R* = Al, Ga, Sc, In, Y, Gd and La) with respect to the R^3+^ ionic radius in Na_2_MnSiO_4_.

We report the solution energies of R_2_O_3_ in Fig. 5. Our calculation reveals that the most favorable dopant solution energy is found for Al^3+^. This suggests that a possible synthesis-doping strategy of introducing additional sodium into Na_2_MnSiO_4_, although the exact amount of Al incorporation cannot be predicted. The possible composition of Al-doped Na_2_MnSiO_4_ would be Na_2+x_MnSi_1−x_Al_x_O_4_ (x = 0.0–1.0). The second most favorable dopant is Ga^3+^. The solution energy increases further with the dopant size.

**Figure 6:**
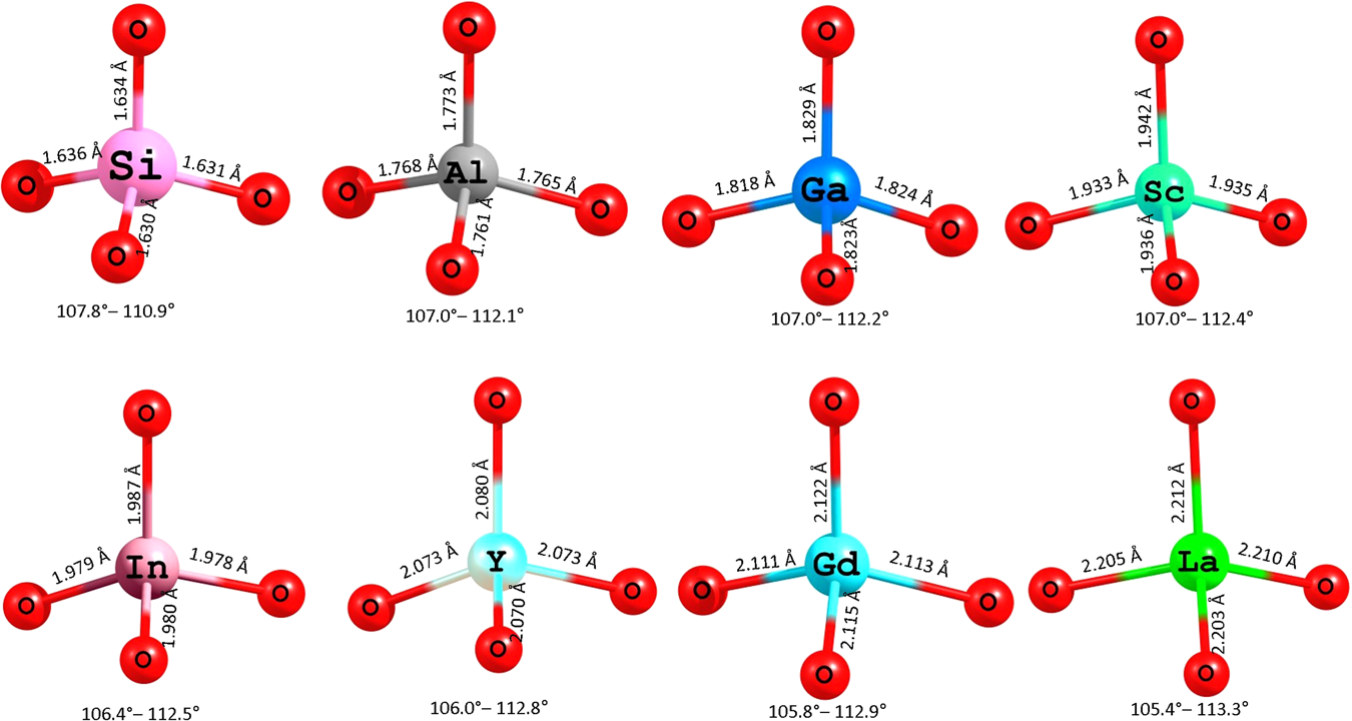
Tetrahedral SiO_4_ unit in the relaxed structure of undoped Na_2_MnSiO_4_ and the coordination formed by the dopants on the Si site with neighbour oxygen.

Figure 6 shows the dopants forming tetrahedral units with adjacent oxygen atoms together with dopant-oxygen bond lengths and oxygen-dopant-oxygen bond angles. For comparison, the tetrahedral SiO_4_ unit in the relaxed structure of defect free Na_2_MnSiO_4_. The ionic radii of Si^4+^ and Al^3+^ in tetrahedral coordination are 0.26 Å and 0.39 Å respectively. The ionic radius of Al^3+^ is 0.13 Å larger than that of Si^4+^. In the SiO_4_ unit, all four Si-O bonds (~1.630 Å) are equal. In AlO_4_ unit, Al-O bond lengths (~1.760 Å) and bond angles are slightly larger than that found in SiO_4_ unit and smaller than that found in other RO_4_ (R = Ga, Sc, In, Y, Gd and La) units. This is reflected in the lowest solution energy for Al. The ionic radius of Ga^3+^ is 0.47 Å slightly larger (by 0.09 Å) than that that of Al^3+^. There is a slight increase in the Ga-O bond lengths and bond angles. This is reflected in the second lowest solution enthalpy. From Sc^3+^ to La^3+^, solution enthalpy steadily increases with ionic radius reflecting in the bond lengths and bond angles. Highly endothermic solution energy values indicate that they will not occur at low temperatures. At elevated temperatures, this process would become feasible.

### Summary

In conclusion, the atomistic simulation techniques were employed to calculate intrinsic defects, sodium ion diffusion paths and trivalent doping in order to assess Na_2_MnSiO_4_ as a promising sodium battery cathode. The dominant energy defect process is Na-Mn anti-site defect suggesting that there would be small intrinsic concentration of Mn on Na sites at operating temperatures. The long range Na ion diffusion path with lowest migration energy was calculated to be three dimensional with the migration energy of 0.81 eV. Solution energies of R_2_O_3_ (R = Al, Ga, Sc, In, Y, Gd and La) were calculated to increase extra Na ions in Na_2_MnSiO_4_. Our calculation suggests that doping Al^3+^ on Si site is an efficient strategy to increase the Na content in Na_2_MnSiO_4_ requiring experimental investigation.

### Computational methods

In the present study we have used the classical pair potential methodology using the GULP package^36^. In essence this is based on the Born model for ionic crystals. The interionic interactions are long-range (attractive: Coulombic) and short-range (repulsive: electron-electron repulsion). For the latter we employed well established Buckingham potentials (Table S1, Supplementary Information)^21,23,37–41^. The relaxation of the atomic positions and lattice parameters was achieved by employing the Broyden-Fletcher-Goldfarb-Shanno (BFGS) algorithm^42^. For the lattice relaxation round defects the Mott-Littleton method^43^ (inner spherical region larger than 700 ions) immediately. Na was placed and fixed at 7 interstitial positions in a linear route between two vacancy sites (all other ions were allowed to relax). Using this series of calculations the maximum energy corresponds to the activation energy of migration along the route. Here we have employed the full charge ionic model with the calculations in the dilute limit. These are sufficient and will correctly calculate trends in energies, however, defect energies are bound to be overestimated^44,45^.

## Electronic supplementary material

Supplementary Information
