# Supplementary material for: Defects, Dopants and Sodium Mobility in Na2MnSiO4
Source: Sci Rep. 2018 Oct 2;8:14669. doi: 10.1038/s41598-018-32856-7 (PMC6168608; doi:10.1038/s41598-018-32856-7)
Supplement: Supplementary file 1 — Supplementary Information [file 41598_2018_32856_MOESM1_ESM.docx]

**Supporting Information**

[**Defects, Dopants and Sodium Mobility in Na_2_MnSiO_4_**](http://ma.ecsdl.org/content/MA2010-03/1/440.short)

Navaratnarajah Kuganathan,^1,a^ and Alexander Chroneos^1,2,b)^

^1^Department of Materials, Imperial College London, London, SW7 2AZ, United Kingdom

^2^Faculty of Engineering, Environment and Computing, Coventry University, Priory Street, Coventry CV1 5FB, United Kingdom

Corresponding authors, e-mails: a) n.kuganathan@imperial.ac.uk

b) [alexander.chroneos@imperial.ac.uk](mailto:alexander.chroneos@imperial.ac.uk)

**Table S1**. Interatomic potential parameters used in the atomistic simulations of Na_2_MnSiO_4_.

Two-body [Φ*_ij_* (*r_ij_*) = *A_ij_* exp (− *r_ij_* /*ρ_ij_*) − *C_ij_ / r_ij_*^6^]

| Interaction | *A* (eV) | *ρ* (Å) | *C* (eV·Å^6^) | Y (e) | K (eV·Å^-2^) |
| --- | --- | --- | --- | --- | --- |
| Na^+^–O^2−[1]^ | 1497.830598 | 0.287483 | 0.00 | 1.000 | 99999 |
| Mn^2+^–O^2−[2]^ | 2601.394 | 0.2780 | 0.00 | 3.420 | 95.0 |
| Si^4+^–O^2–[2]^ | 1283.91 | 0.32052 | 10.66 | 4.000 | 99999 |
| O^2−^–O^2−[2]^ | 22764.30 | 0.1490 | 27.88 | –2.860 | 74.92 |
| Al^3+^ - O^2−[3]^ | 1725.20 | 0.28971 | 0.000 | 3.000 | 99999 |
| Sc^3+^ - O^2−[4]^ | 1575.85 | 0.3211 | 0.000 | 3.000 | 99999 |
| In^3+^ - O^2−[5]^ | 1495.65 | 0.3327 | 4.33 | 3.000 | 99999 |
| Y^3+^ - O^2−[6]^ | 1766.40 | 0.33849 | 19.43 | 3.000 | 99999 |
| Gd^3+^ - O^2−[4]^ | 1885.75 | 0.3399 | 20.34 | 3.000 | 99999 |
| La^3+^ - O^2−[7]^ | 2088.79 | 0.3460 | 23.25 | 3.000 | 99999 |
| Ga^3+^ - O^2−[7]^ | 1625.72 | 0.3019 | 0.000 | 3.000 | 99999 |

**Table S2.** Energetics of intrinsic defect process in Na_2_MnSiO_4_

| Defect process/equation | Reaction energy/eV | Reaction energy per defect/eV |
| --- | --- | --- |
| Na Frenkel /1 | 3.20 | 1.60 |
| Mn Frenkel /2 | 7.48 | 3.74 |
| O Frenkel /3 | 9.84 | 4.92 |
| Si Frenkel /4 | 27.06 | 13.53 |
| Schottky /5 | 43.42 | 5.43 |
| Na_2_O Schottky/6 | 9.75 | 3.25 |
| MnO Schottky/7 | 8.54 | 4.27 |
| SiO_2_ Schottky/8 | 28.54 | 9.51 |
| Na/Mn antisite (isolated) /9 | 1.64 | 0.84 |
| Na/Mn antisite (cluster) /10 | 0.88 | 0.44 |

**References**

1 Treacher, J. C., Wood, S. M., Islam, M. S. & Kendrick, E. Na_2_CoSiO_4_ as a cathode material for sodium-ion batteries: structure, electrochemistry and diffusion pathways. *Phys. Chem. Chem. Phys*, 18, 32744-32752 (2016).

2 Kuganathan, N. & Islam, M. S. Li_2_MnSiO4 Lithium Battery Material: Atomic-Scale Study of Defects, Lithium Mobility, and Trivalent Dopants. *Chem. Mater*. 21, 5196-5202 (2009).

3 Grimes, R. W. Solution of MgO, CaO and TiO_2_ in a-Al_2_O_3_. *J. Am. Ceram. Soc.* 77, 378–384 (1994).

4 Busker, G., Chroneos, A., Grimes, R. W. & Chen, I.-W. Solution mechanisms for dopant oxides in yttria. *J. Am. Ceram. Soc.* 82, 1553-1559 (1999).

5 McCoy, M. A., Grimes, R. W. & Lee, W. E. Planar intergrowth structures in the ZnO–In_2_O_3_ System. *Philos. Mag. A* 76, 1187–1201 (1997).

6 Grimes, R. W. *et al.* The effects of ion size on solution mechanism and defect cluster geometry,” *Ber. Bunden-Ges. Phys. Chem.* 101, 1204–1210 (1997).

7 Tabira, Y., Withers, R. L., Minervini, L. & Grimes, R. W. Systematic structural change in selected rare earth oxide pyrochlores as determined by wide-angle CBED and a comparison with the results of atomistic computer simulation. ‎*J. Solid State Chem*. 153, 16-25, (2000).
